# Effects of tea plant endophytic phosphate-solubilizing bacteria on the growth and selenium uptake of maize seedlings in selenium-rich soil

**DOI:** 10.3389/fmicb.2025.1564159

**Published:** 2025-04-22

**Authors:** Shuqing Zhang, Jinmei Guo, Jianfeng Li, Yousu Yang, Qiuyue Zhang

**Affiliations:** ^1^School of Geography and Resources, Guizhou Education University, Guiyang, China; ^2^Institute of Soil and Environment Bioremediation in Karst Habitats, Guizhou Education University, Guiyang, China; ^3^Key Laboratory of Biological Resources Exploitation and Utilization in Colleges and Universities of Guizhou, Guiyang, China

**Keywords:** phosphate-solubilizing bacteria, biomass, plant height, chlorophyll, nitrogen (N), phosphorus (P), potassium (K), selenium (Se)

## Abstract

Phosphorus (P) is an important nutrient required for plant growth. In this study, seven phosphate-solubilizing bacterial (PSB) strains isolated from tea plant (*Camellia sinensis*) roots were used as test inoculants. After inoculating maize (*Zea mays* L.) seedlings for 45 days, we measured the available AN, AP, AK and available Se contents in the rhizosphere soil, as well as the N, P, K, and Se contents in the plants, along with growth and physiological parameters. The study aimed to explore the effects of endophytic PSB from tea plants on maize seedling growth and the uptake of nutrients such as N, P, K, and Se. The results showed that the endophytic PSB enhanced the P content in maize leaves and the AP content in rhizosphere soil. They also significantly increased seedling fresh weight, dry weight, plant height, and root length. Treatment with *P. fungorum* PMS-05 significantly increased the dry weight, fresh weight, and plant height of seedlings by 103.79, 77.69, and 51.27%, respectively. Inoculation with *P. fungorum* PMS-20 significantly increased plant height and root length by 70.89 and 223.45%, respectively. Furthermore, treatment with *P. fungorum* PMS-05 significantly increased the available Se contents in the rhizosphere soil and the plants by 144.47 and 97.77%, respectively. In conclusion, tea plant endophytic PSB can increase the contents of AP and available Se in rhizosphere soil, promote nutrient absorption, and positively impact seedling growth. *P. fungorum* PMS-05 and *P. fungorum* PMS-20 showed superior growth-promoting effects on seedlings compared with the other strains tested and can be used to develop high-quality PSB agents tailored for crops grown in selenium-rich red soils in Guizhou.

## 1 Introduction

Phosphorus (P) is an essential nutrient element for plants and plays an important role in metabolism (Oldroyd and Leyser, 2020; Isidro et al., 2023). In China, two-thirds of arable land suffers from severe phosphorus deficiency. However, most traditional phosphorus fertilizers applied to the soil are fixed by calcium, iron, and aluminum ions in the soil and thus converted into insoluble phosphates that are difficult for plants to directly absorb and utilize (Chi et al., 2021). Phosphate-solubilizing microorganisms (PSMs) can convert insoluble phosphorus into soluble phosphorus, thereby increasing the availability of phosphorus in the soil and improving the efficiency of phosphorus uptake and utilization by crops (Zhang et al., 2024).

Currently, more than 20 genera of PSM have been identified, including bacteria, fungi, and actinomycetes, with bacteria being the most abundant, followed by fungi and actinomycetes (Chi et al., 2021). Commonly applied phosphate-solubilizing bacteria (PSB) include *Bacillus*, *Burkholderia*, *Azotobacter*, and *Pseudomonas* (Li et al., 2024). PSMs decompose insoluble phosphorus by secreting organic acids, lowering soil pH, and releasing phosphatases. Additionally, they promote crop growth by producing plant growth regulators (Chi et al., 2021). Research has shown that PSB can promote the growth of Chinese cabbage rapa and establish long-term colonization in rhizosphere soil (Wang et al., 2016). In rice, rhizosphere PSB can produce auxins and gibberellins, which exhibit strong nutrient conversion and growth-promoting abilities (Liu et al., 2018). Rhizosphere PSB in camellia plants can enhance the enzymatic activity of leaves and soil, as well as the available phosphorus content in the soil (Xue et al., 2024). PSB in the rhizosphere of maize can produce siderophores, organic acids, zeatin, and auxins, which significantly improve maize plant height, stem diameter, dry weight, and fresh weight (Hou and Hu, 2024). Thus, utilizing PSMs as biofertilizers can enhance fertilizer utilization efficiency, increase crop yields, and improve the soil environment, playing a crucial role in promoting sustainable agricultural development.

In previous studies (Zhang et al., 2024; Guo et al., 2024), after inoculating tea plant endophytic PSB into tea seedlings, it was found that all tested strains significantly increased the available nitrogen (AN) and available phosphorus (AP) content in the rhizosphere soil of “Longjing 43,” enhanced the zinc (Zn) and selenium (Se) content in the aboveground parts of the tea plants, and most strains also improved the available Zn and Se content in the rhizosphere soil. This demonstrated that tea plant endophytic PSB have a positive effect on promoting nutrient absorption in host plants. However, can these strains also promote the growth of non-host plants and improve their nutrient content?

In this study, seven tea plant endophytic PSB were used as the test strains. After inoculating maize seedlings for 45 days, the physiological growth parameters of the seedlings were measured, along with the soil AN, AP, AK and available Se contents and the plant N, P, K, and Se concentrations. The effects of PSB on seedling growth and the uptake of N, P, K, Se, and other nutrients were analyzed. The main goals were: (1) to analyze the effects of PSB on the growth and nutrient uptake of maize seedlings; (2) to evaluate the influence of PSB on rhizosphere soil nutrient dynamics; (3) to compare the differential impacts of tea plant-derived endophytic PSB on growth and nutrient absorption between host (tea) and non-host (maize) plants. This study aims to provide microbial resources and a theoretical foundation for the further development and application of plant growth-promoting bacterial agents.

## 2 Materials and methods

### 2.1 Test materials

#### 2.1.1 Test soil

The soil was collected from the Masson pine-wild oil tea mixed forest in Anping Village, Kaiyang County, Guiyang City, Guizhou Province, China (longitude: 106°59′48.77″, latitude: 27°12′ 10.04′, altitude: 1,086 m). The soil type was red soil. The soil type is red soil. 0-30 cm soil was collected by five-point sampling method and brought back to the laboratory, where impurities were removed, after which the samples were air-dried, homogenized, and prepared for the pot experiment. At the same time, fresh soil samples were analyzed for the total nitrogen (TN), total phosphorus (TP), total potassium (TK), and organic matter contents and soil pH (Zhang et al., 2024; Bao, 2008). The test soil contained 0.40 g⋅kg^–1^ TN, 0.52 g⋅kg^–1^ TP, 4.89 g⋅kg^–1^ TK, 1.44 mg⋅kg^–1^ total Se, and 27.99 g⋅kg^–1^ organic matter and had a pH of 4.6 (Zhang et al., 2024).

#### 2.1.2 Test plant

The maize seeds used in this study were of the variety “Zhongnongtian 488,” purchased from the Cricket Agricultural Technology Extension Service Center (Shouguang City, Shandong Province, China). The seed purity was greater than 95%.

#### 2.1.3 Test strains

The PSB strains (PMS05, PCF06, PSt07, PMS-08, PSt09, PSt12, and PMS-20) were isolated from tea plant roots. These strains were obtained from the Microbial Fertilizer Research Group at the Key Laboratory of Biological Resource Development and Utilization in Guizhou Higher Education Institutions. The strains were isolated from tea plant root tissues collected from tea plantations in Fenggang County (Guizhou Province, China) using PKO (Pikovskaya’s inorganic phosphate-solubilizing medium), Mongina organic phosphate-solubilizing medium, and Winogradsky’s nitrogen-free medium. Based on 16S rRNA sequencing, the strains were identified as *Paraburkholderia fungorum*, *Kluyvera intermedia*, *Paraburkholderia fungorum*, *Paraburkholderia fungorum*, *Enterobacter wuhouensis*, *Kluyvera intermedia*, and *Paraburkholderia fungorum*. Among these strains, PMS05 and PCF06 have dual functions of inorganic phosphorus solubilization and nitrogen fixation; PSt07 and PSt12 can solubilize only inorganic phosphorus; PMS-08 and PMS-20 can solubilize only organic phosphorus; and PSt09 can solubilize both organic and inorganic phosphorus.

#### 2.1.4 Culture medium

LB medium (Liu et al., 2015) was used for cultivation and short-term preservation of the PSB.

### 2.2 Pot Experiment

Maize seeds with full grains and intact seed coats were selected and evenly placed in Petri dishes lined with double-layered filter paper, with 30 seeds per dish and 6 dishes prepared per crop. An appropriate amount of distilled water was added to each dish, and the seeds were placed in a 25°C incubator for uniform germination.

Several flowerpots with a diameter of 18 cm were prepared, and approximately 2,400 g of test soil was added to each pot. Maize seeds with sprouts longer than 1 cm were transplanted into the pots and covered with an appropriate amount of soil. A total of 24 pots were planted, with 4–5 plants per pot. The pots were watered thoroughly the first time, and subsequent watering was adjusted based on weather conditions. The pots were placed outdoors in the experimental cultivation field.

### 2.3 Preparation of PSB suspensions

The seven activated test strains were individually inoculated into LB liquid medium to prepare PSB suspensions. The bottles were sealed with sterile sealing film, labeled, and cultured at 28°C with shaking at 200 r⋅min^–1^ for 20 h. The optical density (OD) of the PSB suspensions at a wavelength of 600 nm was measured using a spectrophotometer. If the OD_600__*nm*_ value was ≥ 1.0, the PSB suspension was transferred to sterile centrifuge tubes and centrifuged at 4,000 r⋅min^–1^ for 10 min. The supernatant was discarded, and an appropriate amount of sterile water was added. The PSB pellet at the bottom of the centrifuge tube was resuspended by shaking, and sterile distilled water was added to adjust the OD_600__*nm*_ of the PSB suspension to 0.8 (Zhang et al., 2024).

### 2.4 PSB inoculation and index measurements

Fifteen days after maize seedling emergence, 100 mL of the prepared PSB suspensions was applied to the root zone of each plant for treatment, with three replicates. The control group received an equal amount of distilled water. After 45 days of inoculation, the entire seedlings were carefully removed, ensuring minimal damage to the roots. The soil attached to the roots was gently shaken off, and soil particles adhering to the root surface were brushed off with a soft brush to obtain rhizosphere soil for this study. The plant height, root length, dry weight, and fresh weight were measured according to the method described by Ke and Pan (2022). The chlorophyll content in the leaves was determined following Wang (2006). The soluble sugar and malondialdehyde (MDA) contents were measured using the thiobarbituric acid method (Zhang, 2004). Root activity was assessed using the TTC method (Zou, 1979). The ammonium nitrogen (NH_4_^+^-N), nitrate nitrogen (NO_3_^−^-N), available phosphorus (AP), and available potassium (AK) contents in the soil and plants were determined according to Bao (2008). The available Se in the soil and the Se content in the plants were measured following the method described by Gou et al. (2013). The available nitrogen (AN) content was calculated as the sum of the ammonium nitrogen and nitrate nitrogen contents. Each sample was analyzed in triplicate.

### 2.5 Data processing

The standard error and average value of three replicates of the test data were calculated. Their one-way ANOVA (analysis of variance) and the Duncan multiple comparison method were implemented using SPSS 23.0 software. Origin 2021 software was used to draw the graphs.

## 3 Results

### 3.1 Effects of tea plant endophytic PSB on fresh and dry weight of maize seedlings

**FIGURE 1:**
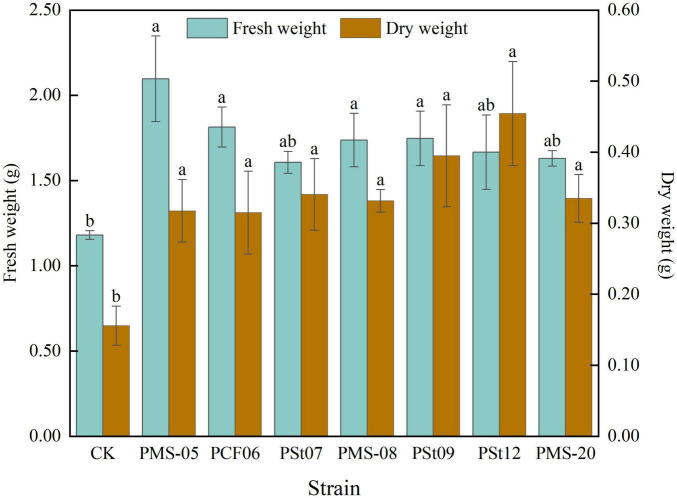
Effects of different strains on fresh and dry weight of maize seedlings. Different lowercase letters in the figure indicate significant differences in maize seedling parameters between different strains (*P* < 0.05). The same applies below.

Inoculation with different PSB strains significantly promoted biomass accumulation in maize seedlings (Figure 1). The fresh weight of the plants in the PSB treatment increased by more than 36.16% compared to the control. The fresh weight of seedlings inoculated with strain PMS-05 was significantly higher than that of the control by 77.69% (*P* < 0.05) and exceeded that of the other PSB treatments by more than 15.63%. Along with the increase in fresh weight, the dry weight of the maize seedlings also increased significantly after PSB inoculation. The increase ranged from 102.12 to 191.78% (*P* < 0.05). Notably, the dry weight of seedlings treated with strain PSt12 reached 202.12% that of the control group (*P* < 0.05).

### 3.2 Effects of tea plant endophytic PSB on the root length and plant height of maize seedlings

**FIGURE 2:**
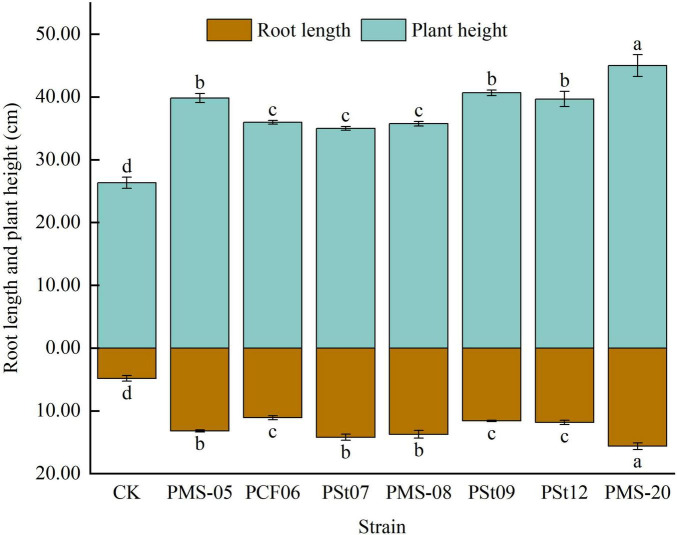
Effect of different strains on root length and plant height of maize seedlings.

Inoculation with PSB significantly increased the root length of maize seedlings by 129.66-223.45% (*P* < 0.05) (Figure 2). Among the tested strains, PMS-20 had the most pronounced effect, with the root length significantly exceeding that of the other treatments by 13.83-40.84% (*P* < 0.05).

Inoculation with PSB significantly increased the plant height of maize seedlings by 13.33-70.89% (*P* < 0.05) (Figure 2). The plant height of seedlings treated with strain PMS-20 reached 170.89% of that of the control and was significantly higher than that of seedlings treated with other PSB strains by 12.97-28.57% (*P* < 0.05). These findings indicate that inoculation with the tested PSB strains can promote the growth of aboveground and underground parts of maize seedlings.

### 3.3 Effects of tea plant endophytic PSB on root activity of maize seedlings

**FIGURE 3:**
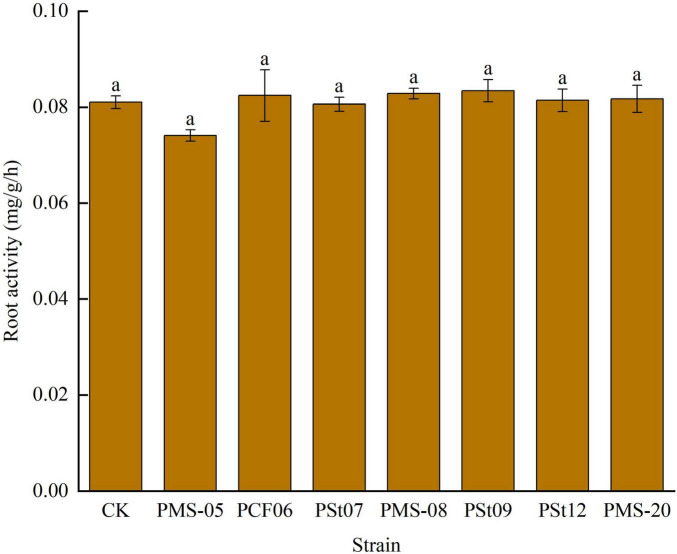
Effect of different strains on root activity of maize seedlings.

After inoculation with different PSB strains, there were no significant changes in root activity of maize seedlings (Figure 3). Only the root activity of seedlings treated with strain PMS-05 was slightly lower than that of the control and the other treatments, but the difference was not significant (*P* > 0.05). These findings suggest that PSB did not cause any damage to the roots.

### 3.4 Effects of tea plant endophytic PSB on chlorophyll content in maize seedling leaves

**FIGURE 4:**
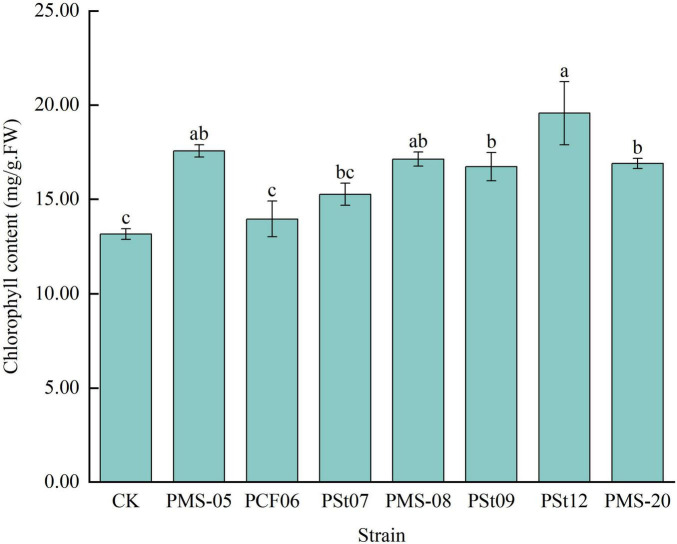
Effect of different strains on chlorophyll content of maize seedling leaves.

The chlorophyll content in maize seedling leaves treated with different PSB strains was 6.08-48.61% higher than that in the control (Figure 4). Among the treatments, strain PSt12 had the most significant effect on increasing the chlorophyll content (*P* < 0.05), which was 39-148.61% of control and other treatments. This strain was followed by strain PMS-05, which results in a chlorophyll content 133.42% that of the control (*P* < 0.05). These results indicate that inoculation with PSB strains can effectively enhance the chlorophyll content in maize seedling leaves, thereby improving photosynthesis in the leaves.

### 3.5 Effects of tea plant endophytic PSB on soluble sugar content in maize seedling leaves

**FIGURE 5:**
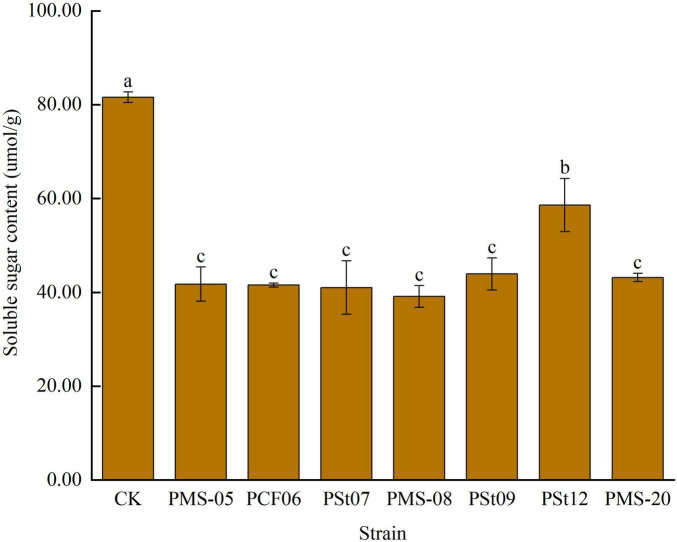
Effects of different strains on soluble sugar content in leaves of maize seedlings.

The rapid accumulation of soluble sugars in leaves is typically a response to mitigate membrane system damage caused by external stresses. The soluble sugar content in maize seedling leaves after inoculation with PSB was significantly lower than that in the control (Figure 5), by 28.17-52.01% (*P* < 0.05). Among the treatments, strain PSt12 caused the smallest change in soluble sugar content. These results suggest that the introduction of PSB as exogenous microbes into the soil temporarily reduced the stress tolerance of maize seedlings.

### 3.6 Effects of tea plant endophytic PSB on malondialdehyde (MDA) content in maize seedling leaves

**FIGURE 6:**
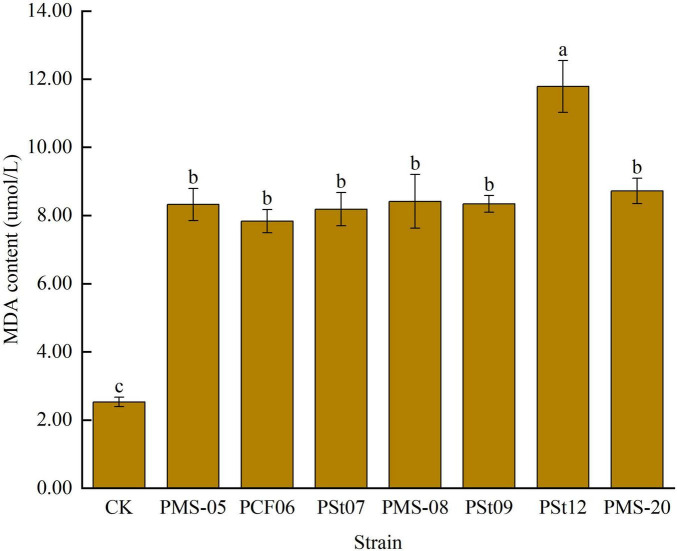
Effect of different strains on MDA content in leaves of maize seedlings.

The MDA content in maize seedling leaves significantly increased under all PSB treatments (Figure 6), with an increase ranging from 209.54 to 365.59% (*P* < 0.05). Among the treatments, strain PSt12 caused the greatest increase in MDA content, indicating that this strain had the most significant effect on the stress tolerance of maize seedlings.

### 3.7 Effects of tea plant endophytic PSB on AN, AP, and AK contents in the rhizosphere soil of maize seedlings

**FIGURE 7:**
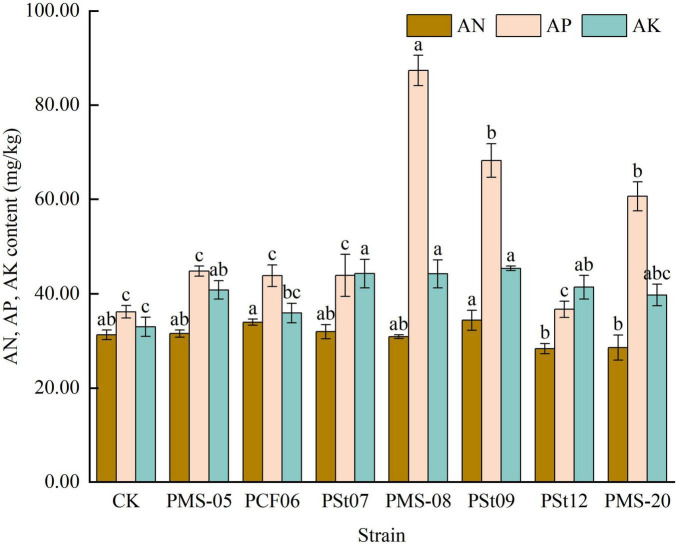
Effect of different strains on the content of AN, AP, and AK in rhizosphere soil of maize seedlings.

After inoculation with PSB, there was no significant change in the AN content in the rhizosphere soil (Figure 7). However, all PSB strains increased the AP content in the rhizosphere soil. Among them, strain PMS-08 significantly enhanced the AP content, which was 27.99-141.47% higher than those in the control and the other treatments (*P* < 0.05). The trend for AK was consistent with that for AP, with all treatments showing increases compared with the control. Strains PSt07, PMS-08, and PSt09 demonstrated the most prominent effects, they were 34.20, 33.99 and 37.58% higher than the control.

### 3.8 Effects of tea plant endophytic PSB on the N P K content of Longjing tea plants

**FIGURE 8:**
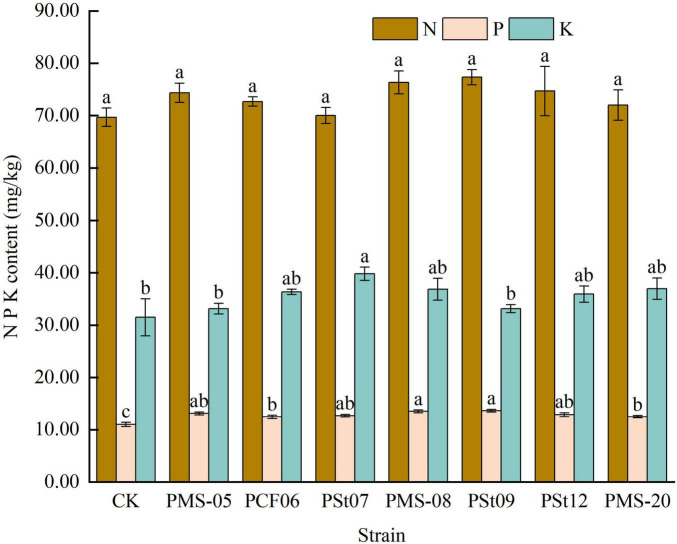
Effect of different strains on N, P, and K contents of maize seedlings.

After inoculation with different PSB strains, the N content in maize seedlings increased slightly but showed no significant differences (Figure 8). The P content increased by 13.33-24.24%, with significant differences observed (*P* < 0.05). Strains PMS-08 and PSt09 were particularly effective at promoting P uptake in seedlings. The K content in the seedlings also increased to varying degrees under all PSB treatments, with strain PSt07 being the most effective in enhancing K absorption, higher than control 26.35%.

### 3.9 Effects of tea plant endophytic PSB on available Se content in rhizosphere soil of maize seedlings

**FIGURE 9:**
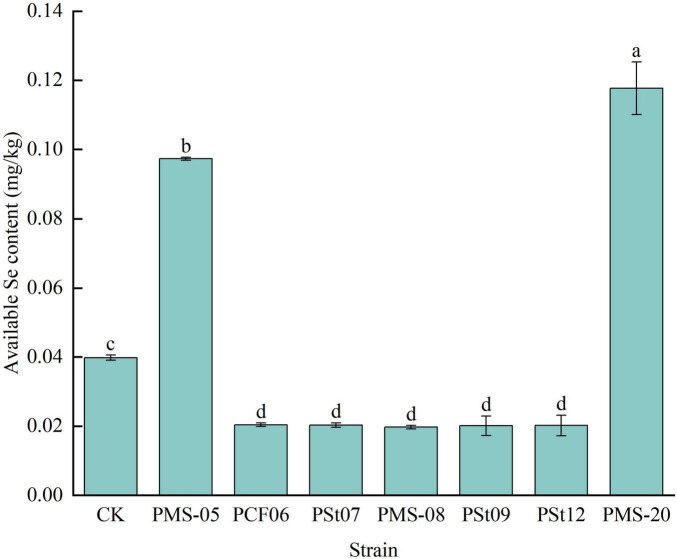
Effect of different strains on the content of available Se in rhizosphere soil of maize seedlings.

Forty-five days after inoculation with different PSB strains, the available Se content in the rhizosphere soil of the maize seedlings showed significant changes (Figure 9). Compared with the control, the rhizosphere soils treated with strains PMS-20 and PMS-05 presented significantly higher available Se contents, with increases of 195.73 and 144.47%, respectively (*P* < 0.05). In contrast, the available Se content under all the other PSB treatments was significantly lower than that of the control (*P* < 0.05).

### 3.10 Effects of tea plant endophytic PSB on Se content in maize seedlings

**FIGURE 10:**
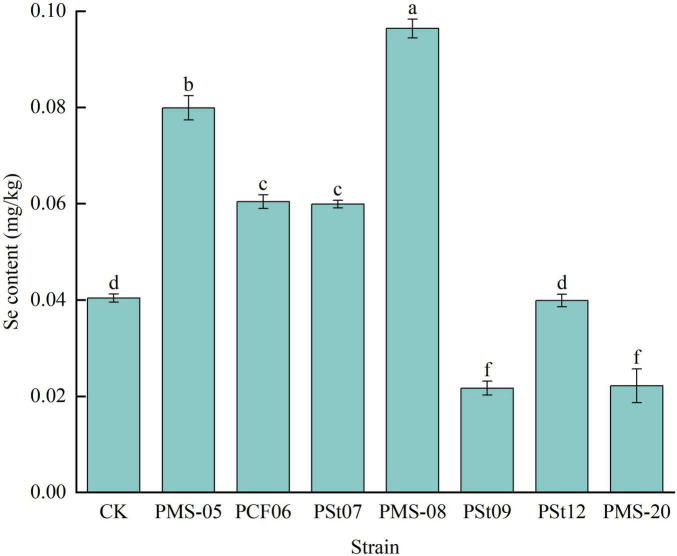
Effects of different strains on Se content of maize seedlings.

The effects of different PSB strains on Se absorption in maize seedlings varied significantly (Figure 10). Four strains noticeably promoted Se uptake in the plants, while two strains inhibited it. Seedlings treated with PMS-05, PCF06, PSt07, and PMS-08 exhibited significant increases in Se content compared to the control, with increases of 97.77, 49.50, 48.27, and 138.61%, respectively (*P* < 0.05). Among these strains, PMS-05 not only significantly increased the available Se content in the rhizosphere soil (Figure 9) but also enhanced the translocation of Se from soil to plants. In contrast, strains PCF06, PSt07, and PMS-08 improved the ability of maize seedlings to absorb and transport Se to leaves, despite reducing the available Se content in the rhizosphere soil.

## 4 Discussion

### 4.1 Effects of tea plant endophytic PSB on the growth and physiology of maize

Exogenous soluble P introduced into the soil is easily adsorbed and fixed by the soil, forming insoluble phosphate compounds. Excessive application of phosphorus fertilizer not only reduces phosphorus use efficiency but also exacerbates soil compaction (Chi et al., 2021). Phosphate-solubilizing and nitrogen-fixing functional microorganisms, as excellent growth-promoting bacterial resources, exhibit strong growth-promoting effects and are environmentally friendly, making them highly promising for development into microbial inoculants. Zhang et al. (2025) found that plant growth-promoting rhizobacteria (PGPR) from the maize rhizosphere can enhance maize biomass accumulation and effectively improve soil nutrient content. Similarly, Hou and Hu (2024) reported that multifunctional growth-promoting bacteria isolated from maize rhizosphere soil significantly promoted the root length, maximum leaf area, chlorophyll content, plant height, stem diameter, dry weight, and fresh weight of maize. Furthermore, Luo et al. (2024) demonstrated that PSB from the maize rhizosphere promoted maize growth to varying degrees and significantly altered the structure of the rhizosphere soil microbial community.

Endophytic bacteria, as one of the sources of PSB, can directly synthesize or promote host-synthesized phytohormones to regulate plant growth (Liu, 2019). Li et al. (2013) demonstrated that PSB isolated from *Anaphalis lactea* significantly enhanced maize growth parameters, including plant height, fresh weight, and dry weight. Wu et al. (2023) demonstrated that inoculation with endophytic fungi increased plant height, stem diameter, leaf number, nitrogen balance index, net photosynthetic rate, transpiration rate of *Camelia Oleifera.* In this study, the phosphorus solubilization and nitrogen fixation effect of the tested strains significantly increased the fresh and dry weight of maize, promoted increases in plant height and root length in maize seedlings, and enhanced chlorophyll synthesis in the leaves, the growth rates were 36.16-77.69%, 102.12-191.78%, 13.33-70.89%, 129.66-223.45% and 6.08-48.61%, respectively.

MDA is the final decomposition product of membrane lipid peroxidation in plant cell membranes under stress, and it serves as a crucial biochemical indicator for evaluating plant tolerance to adversity by reflecting the extent of cellular damage. Soluble sugars, as important osmotic regulators in plants, also play a role in assessing the degree of cell injury (Zhang et al., 2020). Zhou et al. (2022) reported that inoculating mung bean seedlings with PSB isolated from the rhizosphere soils of *Leymus chinensis* and *Suaeda salsa* reduced the MDA content in the seedlings. Similarly, Zhao et al. (2022) found that inoculating *Paris polyphylla* seedlings with PSB at the seedling stage enhanced root activity, increased the soluble sugar content in leaves, and reduced MDA levels. An et al. (2019) demonstrated that inoculating chili pepper seedlings with high-efficiency PSB isolated from the rhizosphere of tobacco significantly increased stem diameter, leaf number, biomass, and soluble protein content but had no significant effect on soluble sugar content. Liu et al. (2024) observed that Bacillus strains increased the soluble sugar content and reduced MDA levels in maize leaves under drought stress conditions.

In this study, maize seedlings were maintained under non-stress conditions without exposure to abiotic stresses such as salinity/alkalinity or drought throughout the experiment. Inoculation with endophytic PSB significantly increased soil available P content, effectively alleviating P deficiency. The enhanced P supply maintained cell membrane integrity, consequently reducing the need for osmotic adjustment through accumulation of compatible solutes like soluble sugars that would otherwise compensate for membrane damage-induced stress (Li et al., 2014). This mechanism explains the observed decrease in soluble sugar content alongside increased MDA levels. An alternative explanation may involve host-specific effects, as the tested PSB strains were originally isolated from tea plant roots, potentially exhibiting differential physiological impacts on host versus non-host plants (Gao et al., 2019).

### 4.2 Effects of tea plant endophytic PSB on the absorption of N, P, and K nutrients in maize

N, P, and K are essential elements for plant growth, with P also being a critical factor influencing photosynthesis and respiration (Liu et al., 2024). Arbuscular mycorrhizal fungi have been shown to promote maize growth and enhance the absorption of N, P, and K by the plant (Chen et al., 2023). Bacillus strains with nitrogen-fixing and phosphate-solubilizing capabilities can also increase the N, P, and K nutrient contents in maize plants (Liu et al., 2024). The contents of nitrate N, AP and organic carbon in rhizosphere soil of *Camelia Oleifera* were increased by inoculation of endophytic fungi (Wu et al., 2023). In previous studies, inoculating host tea plant seedlings with selected PSB strains revealed that PSB could enhance the availability of N, P, and K in the rhizosphere soil of “Longjing 43” tea seedlings (Zhang et al., 2024; Guo et al., 2024). In this study, seven strains of endophytic PSB isolated from tea plants were found to increase the AP and AK contents in rhizosphere soil, while no significant changes were observed in AN content. The effects of different strains on the AN, AP, and AK contents in the rhizosphere soils of host and non-host plants varied. This phenomenon may be due to differences in root exudates between non-host and host plants, which could affect the colonization of the tested strains in the rhizosphere soil, thereby leading to nutrient changes that differ between the two types of plants. Whether these results change with the growth period of the inoculated plants requires further investigation. The increased chlorophyll contents in the maize leaves further indicates that the inoculated PSB promoted the absorption of nutrients by the plants (Liu et al., 2024).

### 4.3 Effects of tea plant endophytic PSB on Se absorption in maize

Se is an essential trace element for humans, and agricultural products are the primary source of Se for the human diet. The Se content in agricultural products largely depends on the available Se content in the soil. PSB can increase the AP content in soil; this AP competes for adsorption sites on soil colloids, activating and releasing SeO_3_^2–^, and consequently increasing the available Se content in soil (Zhang et al., 2024). Durána et al. (2015) reported that endophytic bacteria could significantly increase the Se content in wheat crops.

In a separate experiment, inoculation of the tested strains into host tea plant seedlings revealed that five strains of PSB significantly increased the available Se content in the rhizosphere soil and the Se content in tea plant roots. Among them, the PSt12 strain notably increased the available Se content in the rhizosphere soil and Se content in tea plant roots by 573.07 and 397.26%, respectively, compared to the control (*P* < 0.05) (Zhang et al., 2024; Guo et al., 2024). However, in this study, the PSt12 strain had no significant effect on the Se content in maize leaves and even significantly reduced the available Se content in the rhizosphere soil of maize. These findings suggest that, as an endophyte originating from tea plant tissues, the strain has different effects on Se absorption and transport in host and non-host plants. This discrepancy may also be attributed to the differences in nutrient uptake characteristics between tea plants and maize. Additionally, the Se content in the aboveground parts of maize is influenced by soil texture, plant variety, and growth stage (Placzek and Barbara, 2020).

## 5 Conclusion

In this study, seven strains of endophytic PSB isolated from tea plant roots were inoculated into maize seedlings to analyze their effects on maize seedling growth, physiology, and soil nutrients. The results showed that all seven strains of tea plant endophytic PSB significantly increased the fresh weight, dry weight, plant height, and root length of maize seedlings. Fresh weight increased the most under *P. fungorum* PMS-05 treatment, plant height and root length increased the most under *P. fungorum* PMS-20 treatment. All strains enhanced the AP content in the rhizosphere soil and the P content in the maize plants. Specifically, treatment with *P. fungorum* PMS-05 significantly increased the available Se content in the rhizosphere soil and the Se content in the maize plants (*P* < 0.05).

In conclusion, tea plant endophytic PSB promote the growth of maize seedlings, improve rhizosphere soil nutrients, and enhance nutrient uptake by plants. *P. fungorum* PMS-05 and *P. fungorum* PMS-20 can be considered potential growth-promoting bacterial resources for the early growth stages of maize.

## Data Availability

The original contributions presented in this study are included in this article/supplementary material, further inquiries can be directed to the corresponding author.
